# Supplementary material for: Reliability of Dynamic Shoulder Strength Test Battery Using Multi-Joint Isokinetic Device
Source: Sensors (Basel). 2024 Jun 1;24(11):3568. doi: 10.3390/s24113568 (PMC11175324; doi:10.3390/s24113568)
Supplement: Supplementary file 1 [file sensors-24-03568-s001.zip › sensors-3006154-supplementary.pdf]

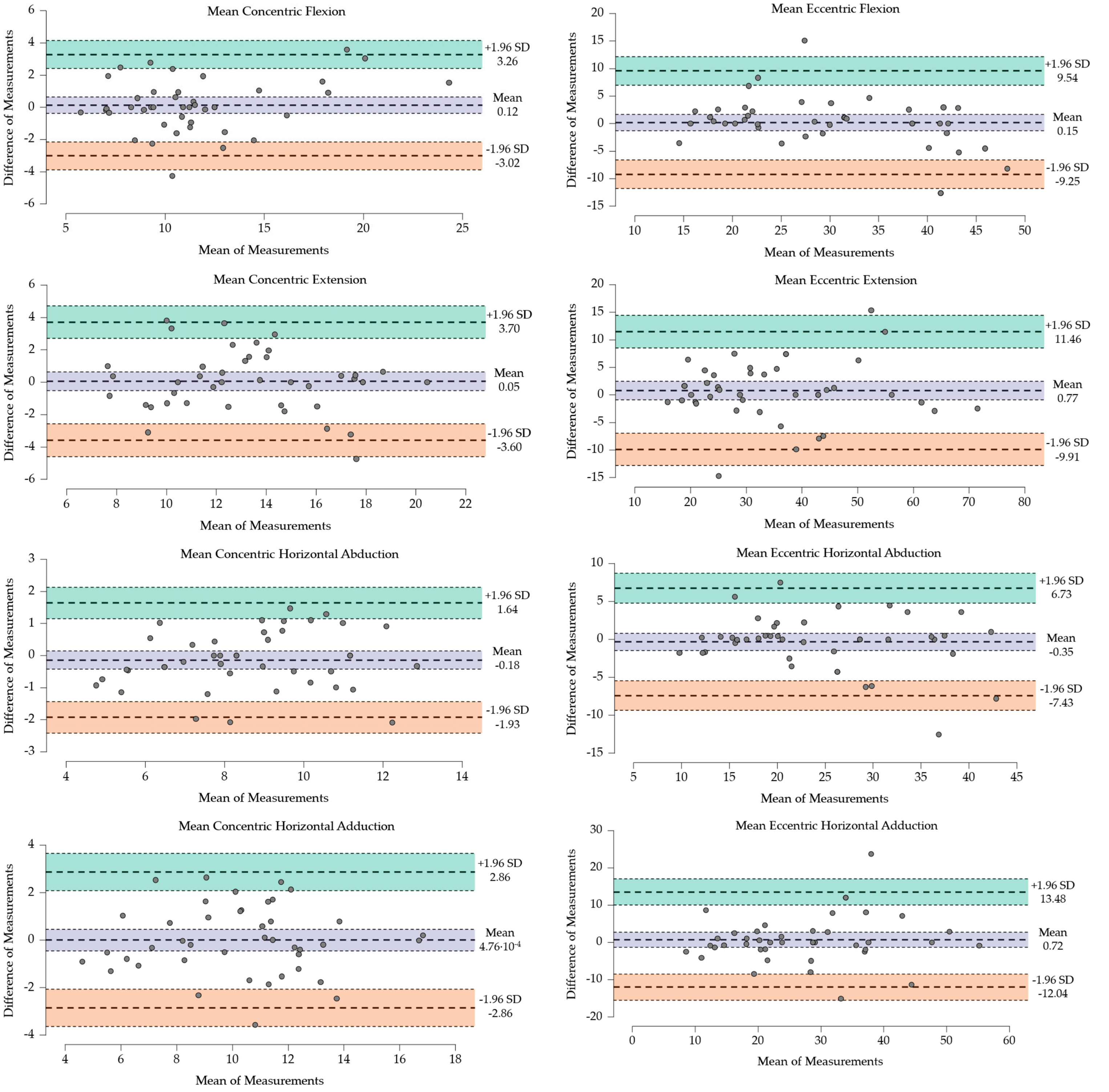

Supplementary material Figure S1. Bland-Altman plots for concentric and eccentric mean force

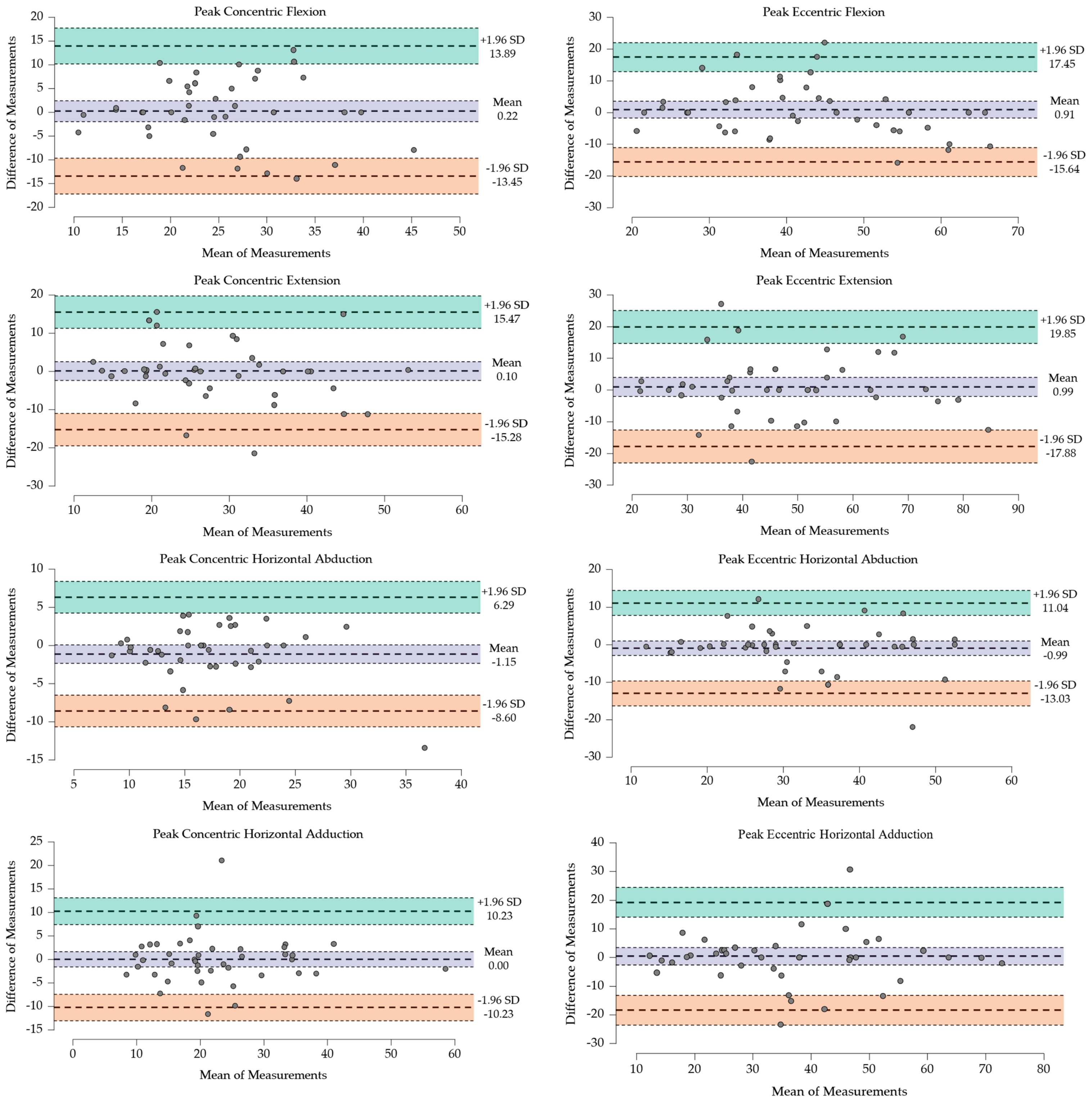

Supplementary material Figure S2. Bland-Altman plots for concentric and eccentric peak force
